# The profiling of pre- and post-warming DNA in mouse embryos with microsatellite method

**DOI:** 10.14202/vetworld.2018.1526-1531

**Published:** 2018-11-02

**Authors:** Widjiati Widjiati, Soeharsono Soeharsono, Yeni Dhamayanti

**Affiliations:** Department of Veterinary Anatomy, Faculty of Veterinary Medicine University of Airlangga, Surabaya, Indonesia

**Keywords:** blastocyst, DNA mutation, single-strand conformation polymorphism, vitrification

## Abstract

**Aims:**

This research aimed to identify the deoxyribonucleic acid (DNA) profile and changes of post-warming embryo after being frozen with vitrification method using microsatellite method.

**Materials and Methods:**

This research examined the mouse embryo blastocysts that were divided into four groups: Post-warming living blastocyst, post-warming living blastocyst with half fragmented cell, post-warming dead blastocyst, and pre-freezing living blastocyst. The isolation sample applied phenol-chloroform method. After obtaining polymerase chain reaction results, all the samples of pre-freezing fresh embryo, post-warming living embryo, dead embryo, and degenerated embryo were then examined by single-strand conformation polymorphism (SSCP).

**Results:**

The amplification with D18mit14 primer was 100 bp and 150bp with D18mit87 primer, 150bp with D7mit22, and 300bp with D7mit25. The result of SSCP with D18mit14 primer showed that the blastocysts were fragmented and dead after warming process and formed into two DNA strand fragments, while the fresh embryos which passed freezing process did not form any fragment. D18mit87 primer SSCP indicated different fragments for each treatment. The result of SSCP using D7mit22 formed two different fragments for each treatment. While using D7mit25, the SSCP result formed some different fragments for each sample. Post-warming living embryo had similar ribbon to pre-freezing fresh embryo.

**Conclusion:**

D7mit222, D7mit25, and D18mit87 primers could be used as the aneuploidy marker on mouse embryos that were induced by post-warming process. The profile of living blastocyst, dead blastocyst, and post-warming fragmented blastocyst had different DNA tapes.

## Introduction

*In vitro* fertilization is one of the common techniques in assisting reproduction. Embryo transfer to the uterus is possible at all stages, but blastocyst stage has the highest pregnancy rate compared to the other stages. Blastocyst stage transfer could be considered as a selection process to prevent genetic disorders and to obtain high-quality embryo. The embryonic dormancy is usually caused by aneuploidy or abnormal numbers of chromosome [1]. The limiting factors in blastocyst stage transfer are freezing and storing the blastocyst. When embryo is being transferred to the uterus, the other embryo should be stored to be used in another cycle or functioned as a donation to the infertile couple [2].

Freezing is an effective way to preserve embryo obtained from frozen *in vitro* fertilization and will stop the metabolism process [3]. Vitrification is one of the methods to cryopreserve embryo. One of the main advantages from vitrification is the absence of crystallization that causes cell damage [4-7], whereas, in the other freezing methods, the crystallization occurs. To avoid crystallization damaging in the blastomere cell in the vitrification, this method needs the use of high concentration cryoprotectant [8,9].

The problem in this method is the low quality of post-warming embryo [9] because embryos are exposed to the high-stress level [10]. It can be seen if post-warming embryo is cultured and transferred into the recipients with low pregnancy number. The low number of implantation and pregnancy is influenced by many factors such as the number of blastomere cells, trophoblast, and also inner cell mass which were degenerated. Thus, it will decrease the viability of embryo and implantation number [11]. During the warming process, there is a temperature change from cold to warm, and this condition forms free radicals. These free radicals can damage the trophoblast and inner cell mass and finally change the structure of nucleotides on the embryo’s deoxyribonucleic acid (DNA) [12]. The cell needs DNA to survive and prevent the unstable genomic that can cause death and cellular changes. Oocyte or mammals’ embryo is vulnerable to DNA damage because DNA is the basic component for all proteins and macromolecules that carry genetic information [13-16]. Each cell has a different basic structure, and each organelle contains genetic material. The changes in DNA basic structure can cause different genetic code [17-19].

Single-strand conformation polymorphism (SSCP) is the simplest method to detect DNA mutation. SSCP is widely used to detect the variation of DNA sequence which can identify the new mutation, so it can be used to discover defect genetic. This method will separate DNA from amplification using single-strand polymerase chain reaction (PCR) by electrophoresis on the non-denaturing polyacrylamide gel [20].

The research objectives were to identify the DNA profile of post-warming embryo after being frozen with vitrification method and to reveal the DNA changes of post-warming mouse embryo using microsatellite method to confirm the effect of vitrification process toward chromosomal disorder or aneuploidy.

## Materials and Methods

### Ethical approval

The ethical clearance certificate number 718-KE was obtained from the Faculty of Veterinary Medicine Universitas Airlangga, Surabaya, Indonesia for the current study.

### Materials

This experimental study was conducted at the Experimental Animal Laboratory of the Faculty of Veterinary Medicine in Airlangga University, from February 6, 2017, to April 15, 2017. A total of 30 female mice, strain Balb/C, 3 months old, weight ranged from 30 g to 35 g, and 30 male mice, strain Balb/C, 4 months old, weight ranged from 40 g to 45 g, were collected from Veterinary Pharmacy Center, Surabaya, Indonesia. The oocytes were attained by ripping the fertilization sac under an inverted microscope using a 27-G needle and iris forceps to handle the fallopian tube. The sperms were collected from male mice cauda epididymis.

The materials utilized on this research were pregnant mare serum gonadotropin (PMSG) (Folligon^®^, Intervet, Boxmeer, Holland), human chorionic gonadotropin (Chorulon^®^, Intervet, Boxmeer, Holland), phosphate buffer saline (PBS), Dulbecco’s Medium Eagle’s Medium (Sigma^®^, St. Louis, USA), ethylene glycol (Sigma^®^, St. Louis, USA), propanediol (Sigma^®^, St. Louis, USA), mineral oil (Sigma^®^, St. Louis, USA), gentamicin sulfate, CO_2_, lysis buffer to isolate the DNA, protease enzyme, constriction enzyme, chloroform (Merck), phenol (Merck), isoamyl alcohol (Merck), EDTA (Bioworld), PCR mix (Go Taq green Promega), primer (integrated DNA technologies), NaOH (Merck), bromophenol blue (Nacalai Tesque), urea (Promega), boric acid (Bioworld), SDS (Bioworld), DTT (Nacalai Tesque), agaroses (intron), formalin (Merck), silver nitrate (SAS), marker DNA 10kb (Intron), tetramethylethylenediamine (TEMED) (Wako), and APS (Nacalai Tesque).

The instruments of this research were CO_2_ incubator (Thermo), inverted microscope (Nikon), real-time PCR, Tris (Bioworld), incubator (Memmert), vortex (Vortex mixer), centrifuge (Hettich), ethanol (Merck), acrylamide 30% (Nacalai Tesque), PCR machine (Takara), Tube 1.5 ml (Stardeck), vertical electrophoresis chamber (Bio Craft), PCR tube (Biologix), UV transilluminator (Bio-Rad), and spin-down (Force Mini).

### Methods

#### Sample preparation, in vitro fertilization, embryo culture, and vitrification

The female mice were induced with PMSG (Folligon) with a dosage of 5 IU and followed by 5 IU human chorionic gonadotropin (Chorulon) injection after 48 h. The female mice were mated with vasectomized male mice to induce ovulation. After 17 h, the oocyte collection was executed by ripping the fertilization sac under the inverted microscope using a 27-G needle and iris forceps to handle the fallopian tube. The oocytes were then collected using modified pipette [21].

After that, the collected oocytes were cleaned by putting the oocytes into fertilization medium (MEM). The cleaned oocytes were put into the *in vitro* fertilization medium that had been incubated a day before. Next, the oocytes were incubated at 37°C. A midline laparotomy was performed to expose the cauda epididymis. The cauda epididymis was previously cleaned, cut up, and diluted in saline solution. The spermatozoon was collected with the dosage of 300,000 and put into a medium filled with oocytes using modified sperm pipette before incubated in 5% CO_2_ at 37°C [22].

The formed zygotes were moved into a culture medium. On the day 2^nd^ and day 4^th^, the culture medium was changed to give enough nutrition and to support the embryo development in blastocyst stage [21].

The blastocysts were collected together and then randomly divided into four groups: Post-warming living blastocyst, post-warming living blastocyst with half fragmented cells, post-warming dead blastocyst, and pre-warming living blastocyst. The blastocysts were cryopreserved with vitrification method using Hemi-Straw. The cryoprotectant used in this research was the combination of ethylene glycol and insulin transferrin selenium. The blastocyst-filled hemi straw was first placed above the evaporation of N2 liquid. It was then dipped in N2 liquid and packed in a big straw. The straw tip was fixated so that the hemi straw stayed orderly. The big hemi straw was placed into a straw disk and goblet container with N2 liquid [23].

#### Embryo warming

The cryopreserved blastocysts were warmed after being put into a medium with V4 (PBS medium+0.5 M sucrose) for 2.5 min and V5 (PBS medium + 1M sucrose) for 7.5 min gradually [22].

#### DNA isolation

The samples were isolated by phenol-chloroform method. The samples were added with buffer lysis containing 100 mM Tris-Cl pH 7.5, 50 mM Na2EDTA, 0.5% SDS, and 1 mM DTT and incubated at 55°C overnight. Those were also added with 300 µL PCI (phenol: chloroform: isoamyl alcohol with ratio 25:24:1) and put on a vortex for 10 s before being centrifuged in 10,000 rpm for 10 min at room temperature. After the centrifugation, the samples formed two layers: The first layer consisted of DNA liquid and the second layer consisted of organic PCI solvent. The first layer was then moved into a 1.5 mL new tube and added with CI (chloroform: isoamyl alcohol with ratio 24:1). Next, the samples were placed on a vortex for 10 s and centrifuged in 10,000 rpm for 10 min at room temperature. There were two layers in a 1.5 mL tube. The first layer proceeded into a new 1.5 mL tube, then added with 1000 µL cold absolute ethanol, and shook slowly. The samples were incubated at −20°C for an hour and then centrifuged in 10,000 rpm for 10 min at 4°C. After throwing the absolute ethanol, the samples were added with 70% 500 µL ethanol and centrifuged in 10,000 rpm for 10 min at 4°C. The absolute ethanol was taken out again from the samples. The tubes containing DNA were dried at 55°C overnight and added with 60 µL Tris-EDTA pH 7.5. Those were also incubated at 55°C for 3 h [24].

#### DNA amplification using PCR

The DNA samples were later proceeded into PCR using mitochondria primer; D7Mit25 (5’-AGG GGC ACA TGT TCA ACT ATG-3’ and 5’-GGT TGT TTC CAG CTT TGGG-3’) primer, D7Mit222 (5’-AGT GCA GGG AGA AAA CCT GA-3’ and 5’-CAA AAA TGT TGT CTT AAG TTT ATG TGG-3’) primer, D18Mit14 (5’-GAG GTG ATG TGG ACA CAC TC-3’ and 5’-ACA CAG CCT AGA ATG CAC GG-3’) primer, and D18Mit87 (5’-TAT TAA AAG TTC ATT TGT GCA TGT ACA-3’ and 5’-ACT GGG AAA AGT ACC ACT GTA AGG-3’) primer. The compositions of PCR were 2 µL ddH_2_O, 2 µL primer with 10 µM concentration, 5 µL 2× GoTaq Green Master Mix (Promega Corporation), and 1 µL DNA. Next, the compounds were spun down and inserted into PCR machine with 5-min pre-denaturation program at 95°C, 35 denaturation cycles at 95°C in 30 s, 30-min annealing at 55°C, and 30-s extension at 72°C. The final extension was done in 10 min at 72°C [24].

### The making of agarose gel

First, 0.060 g agarose was added with 30 mL Tris-Boric acid-EDTA (TBE) buffer pH 8.3 and heated in a microwave until being dissolved. Second, the compounds were added with 2 µL ethidium bromide. Next, the DNA liquid was poured into a gel box with well combs. After the gel was formed, the plate containing gel was put into an electrophoresis chamber. The samples were placed in every well comb, and DNA marker was placed into one of the wells. After the running process was done, the gel was put onto transilluminator and photographed with a digital camera [24].

### SSCP

After the PCR result was obtained, all the samples of pre-freezing fresh embryo, post-warming living embryo, dead embryo, and degenerated embryo were then examined using SSCP. The amplification results were proceeded using denaturing urea polyacrylamide gel electrophoresis [25] with D7Mit222, D18Mit14, 12% D18Mit14, and 10% D7mit25 primers. 10% gel consisted of 6 g urea, 2.5 mL acrylamide 30%, 0.75 mL TBE 10×, 1.5 mL aquadest, 100 µL APS 10%, and 5 µL TEMED. 12% gel contained 3.6 g urea, 3.125 mL acrylamide 30%, 0.75 ml TBE 10×, 0.875 mL aquadest, 100 µL APS 10%, and 5 µL TEMED. The compounds were moved into a vertical gel box installed with well comb and proceeded until forming a gel. The results from PCR sample were added with 5 µL 95% formamide, 10 mM NaOH, 0.05% bromophenol blue, and 20 mm EDTA. Those were then heated at 100°C for 5 min before being placed into a refrigerator. Finally, the samples were put into well comb and proceeded at 190 V during 4.5 h.

### Silver nitrate coloring

The gel from SSCP processing was then colored with silver nitrate [26]. It was fixated with 10% ethanol and 0.5% glacial acetate acid during 30 min. The gel was then colored with 1.5 g/L and 100 µL silver nitrate during 30 min. Next, the gel was washed with aquadest for 5 min and poured with 1.5 g/L and 100 µL NaOH until forming tapes. The gel was then scanned and analyzed.

### Results

**Figure-1:**
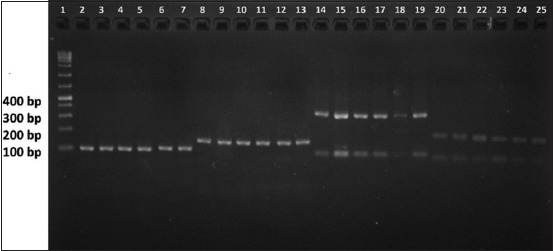
The polymerase chain reaction results using D18mit14, D7mit222, D7mit25, and D18mit87 primer. 1. Marker 1 kb; 2. Live zygote (D18mit14); 3. Post-warming living embryo (D18mit14); 4. Fragmented embryo (D18mit14); 5. Post-warming dead embryo (D18mit14); 6. Post-warming dead embryo (D18mit14); 7. Pre-freezing fresh embryo (D18mit14); 8. Live zygote (D7mit222); 9. Post-warming living embryo (D7mit222); 10. Fragmented embryo (D7mit222); 11. Post-warming dead embryo (D7mit222); 12. Post warming dead embryo (D7mit222); 13. Pre-freezing fresh embryo (D7mit222); 14. Live zygote (D7mit25); 15. Post-warming living embryo (D7mit25); 16. Fragmented embryo (D7mit25); 17. Post-warming dead embryo (D7mit25); 18. Post-warming dead embryo (D7mit25); 19. Pre-freezing fresh embryo (D7mit25); 20. Live zygote (D18mit87); 21. Post-warming living embryo (D18mit87); 22. Fragmented embryo (D18mit87); 23. Post warming dead embryo (D18mit87); 24. Post warming dead embryo (D18mit87); 25. Pre-freezing fresh embryo (D18mit87).

The amplification result with D18mit14 primer was about 100 bp and 150 bp with D7mit222 primer, 300 bp with D7mit25 primer, and 150 bp with D18mit87 primer. The amplification result variety for each primer depends on the length of guanine-timing replication on nucleotides at D7mit25 and the length of cytosine-adenine replication on nucleotides at D7mit22, D18mit14, and D18mit87. The longer the replication area, the longer the primer amplification result. The PCR results are shown in Figure-1.

**Figure-2:**
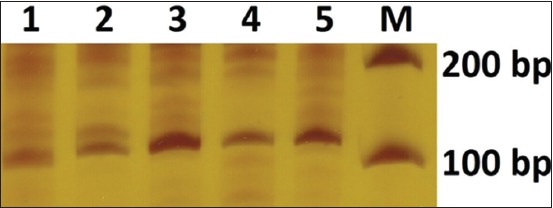
The result of single-strand conformation polymorphism D18mit 14; 1. Post-warming living blastocyst; 2. Half-fragmented post-warming living blastocyst; 3. Post-warming dead blastocyst; 4. Post-warming dead blastocyst; 5. Pre-freezing living blastocyst.

The SSCP result with D18mit14 primer is shown in Figure-2. The SSCP result with D18mit14 primer shows that there were two DNA-strand fragments formed in fragmented embryos. Post-warming embryo in samples 3 and 4 causes the establishment of fragmented tapes as informed in the below section. Sample numbers 1 and 5 prove that pre-freezing fresh embryo process does not form any fragment.

**Figure-3:**
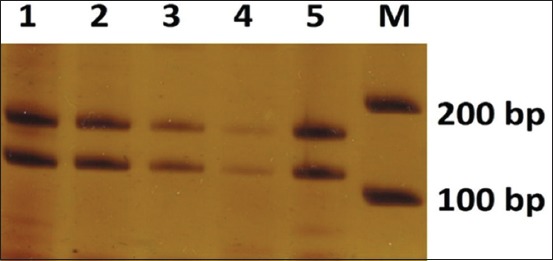
The result of single-strand conformation polymorphism D7mit222; 1. Post-warming living blastocyst; 2. Half-fragmented post-warming living blastocyst; 3. Post-warming dead blastocyst; 4. Post-warming dead blastocyst; 5. Pre-freezing living-blastocyst.

**Figure-4:**
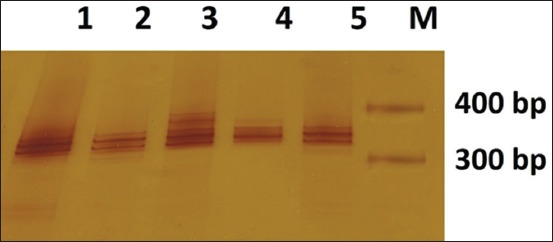
The result of single-strand conformation polymorphism D7mit25; 1. Post-warming live blastocyst; 2. Half-fragmented post-warming live blastocyst; 3. Post-warming dead blastocyst; 4. Post-warming dead blastocyst; 5. Pre-freezing live blastocyst.

**Figure-5:**
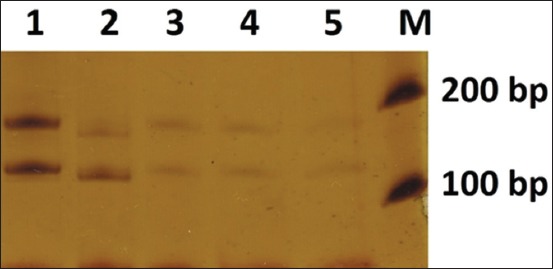
The result of single-strand conformation polymorphism D18mit87; 1. Post-warming living blastocyst; 2. Half-fragmented post-warming living blastocyst; 3. Post-warming dead blastocyst; 4. Post-warming dead blastocyst; 5. Pre-freezing living blastocyst.

The result of SSCP using D7mit222 marker is shown in Figure-3. It presents the results of SSCP D7mit222 which forms two fragments on each sample. The results of SSCP with D7mit25 marker are shown in Figure-4, indicating that each sample forms different fragments. The results of SSCP with D18mit87 marker can be obtained in Figure-5. Figure-5 shows that each sample forms different fragments. Post-warming living embryo forms different tapes compared to the other samples. The tapes are thinner in the below segment, whereas post-warming dead embryo and pre-freezing fresh embryo have similar tapes.

## Discussion

The main problem of pre-freezing blastocyst is the liquid composition in blastocoel cavity. This cavity causes a limitation to freeze substance access in the inner cells and can cause the formation of crystal ice [27]. To avoid the formation of crystal ice, a high concentration cryoprotectant is needed. High concentration cryoprotectant can harm the embryo and may lead to cellular damage [28]. Former studies showed that vitrification process and warming process have damaging effects on the inner and outer cell mass [1].

SSCP is one of the simple screening techniques for detecting the formation of unknown mutation, such as single-basic substitution, small detection, small insertion, or microinversion. The various DNAs generate alteration on the fragmented DNA formation in the electrophoresis gel. Almost 80-90% of mutations can be detected using SSCP [29].

Aneuploidy is an abnormal number of chromosome. The chromosome mutation induces aneuploidy. Aneuploidy is different from wild type. The numbers of chromosome in wild type can be more or less. The microsatellite is the simple marker of the basic nucleotide replication in mammal’s genome and can be used to detect the defected locus and segregation pattern. Each strain, especially in mice, has different nucleotide replication. This replication can occur during recombination or replication. In this area, mispairing and slipping potentially occur. The chromosome 7 markers which are D7mit25 and D7mit222 can be used as the first aneuploidy marker. Meanwhile, the chromosome 18 markers which are D18mit14 and D18mit87 can be used as second marker to confirm if there is ambiguity on the first marker [7]. D7mit222 is a primer designed from chromosome 7 segment with full DNA segment name, Chr7, Massachusetts Institute of Technology 222.

Microsatellite marker is derived from genome’s simple sequence repeat in genetic information examination. This method is used to identify locus defective mapping. Toward the mice identified to have 10,000 cytosine-adenine (CA), the average repetition was one locus per 30 kb. The repetition occurred during recombination or replication and mispairing in this area. The variation of CA repetition length was discovered among different mice’s strain [25].

This study has found that the pre-freezing fresh embryo and post-warming living blastocyst have similar tapes. This means that vitrification does not form any aneuploidy.

## Conclusion

The conclusion of this research is that D7mit222 primer, D7mit25 primer, and D18mit87 primer can be used as the aneuploidy markers on mouse embryos induced by post-warming process. Post-warming living embryo has similar tape to pre-freezing fresh embryo’s tape. It can be assumed that fresh embryo can survive after warming process.

## Authors’ Contributions

WW, SS, and YD had the original idea for the study and carried out the design. WW and SS were responsible for data acquisition. SS was responsible for analysis and interpretation of the data. YD was responsible for drafting and revising the manuscript critically for important intellectual content. All authors read and approved the final manuscript.
